# Ensuring the right to food for indigenous children: a case study of stakeholder perspectives on policy options to ensure the rights of tamariki Māori to healthy food

**DOI:** 10.1186/s12939-021-01407-4

**Published:** 2021-02-27

**Authors:** Christina McKerchar, Cameron Lacey, Gillian Abel, Louise Signal

**Affiliations:** 1 Department of Population Health, University of Otago, PO Box 4345, Christchurch, 8140 New Zealand; 2 Māori/Indigenous Health Institute, University of Otago, PO Box 4345, 8140 Christchurch, New Zealand; 3 Department of Public Health, University of Otago, PO Box 7343, Wellington, South Wellington 6242 New Zealand

**Keywords:** Children’s rights, Indigenous, Food policy, Food security, Obesity

## Abstract

**Background:**

The United Nations Convention on the Rights of the Child confirms a child’s right to adequate food, and to the highest attainable standard of health. For indigenous children, these rights are also recognised in the UN Declaration on the Rights of Indigenous Peoples. However, Indigenous children endure higher rates of obesity and related health conditions than non-indigenous children, including in Aotearoa New Zealand (NZ). For indigenous tamariki (Māori children) in NZ, high levels of obesity are interconnected with high rates of food insecurity. Therefore there is a need for action. This study aimed to investigate policy options that would safeguard the rights of indigenous children to healthy food. We explored with key stakeholder’s policy options to ensure the rights of indigenous children to healthy food, through a case study of the rights of tamariki.

**Methods:**

Interviews were conducted with 15 key stakeholders, with experience in research, development or delivery of policies to safeguard the rights of tamariki to healthy food. Iterative thematic analysis of the transcripts identified both deductive themes informed by Kaupapa Māori theory and literature on rights-based approaches and inductive themes from the interviews.

**Results:**

The analysis suggests that to ensure the right to adequate food and to healthy food availability for tamariki, there needs to be: a comprehensive policy response that supports children’s rights; an end to child poverty; food provision and food policy in schools; local government policy to promote healthy food availability; and stronger Māori voices and values in decision-making.

**Conclusions:**

The right to food for indigenous children, is linked to political and economic systems that are an outcome of colonisation. A decolonising approach where Māori voices and values are central within NZ policies and policy-making processes is needed. Given the importance of food to health, a broad policy approach from the NZ government to ensure the right to adequate food is urgent. This includes economic policies to end child poverty and specific strategies such as food provision and food policy in schools. The role of Iwi (tribes) and local governments needs to be further explored if we are to improve the right to adequate food within regions of NZ.

## Background

The United Nations Convention on the Rights of the Child recognises a child’s right to adequate food, and to the highest attainable standard of health [1, 2]. In the case of indigenous children, the UN Declaration on the Rights of Indigenous Peoples [3], also recognises the right to environments that enable access to traditional foods. Many indigenous peoples have experienced a nutrition transition, with the introduction of a westernised diet and lifestyle, which has affected their right to food and contributed to the development of obesity [4]. In many high-income countries, including Australia, Canada and USA, there is marked inequity between the rates of obesity for indigenous children compared to non-indigenous children [5–7]. Obesity is classified as a form of malnutrition. Although malnutrition has been traditionally associated with undernutrition, since the 1980s obesity has increased rapidly in high-income countries, and is often associated with mild to moderate food insecurity [8]. Obesity is a risk factor for non-communicable diseases, including cardiovascular disease, type 2 diabetes, various cancers, and depression [9–11]. Obesity in childhood can expose a child to a form of prejudice termed weight bias, which increases the risk of stigma and discrimination [12].

In NZ in the past 15 years, obesity rates have increased from 8.4% of children in 2006 to 11.3% in 2018 [13]. While obesity [8] rates have increased in children of all ethnicities, prevalence is high for indigenous tamariki at 15.5% in 2018/19 [13], which is associated with socioeconomic inequity. Children living in the most deprived areas of NZ are 2.7 times as likely to be obese as children living in the least deprived areas [13]. NZ has an inequitable distribution of wealth by ethnicity, especially marked since economic changes introduced since the 1980s [14]. The number of tamariki living in relative poverty was estimated to be one in four children (69,100) in the year ending June 2019 [15]. The reality of this is that almost a quarter of tamariki (23.3%) lived in households doing without basic material needs, including fresh fruit and vegetables or meals that include meat, fish or chicken [16]. These figures correlate with research from 2015/16 that found over one in four (28.6%) tamariki lived in food-insecure households [17]. Due to the recent economic impacts of Covid-19, it is likely that poverty and food insecurity have both increased.

To uphold the right to adequate food, healthy food needs to be available. Food availability is a concept that refers to the adequacy of the supply of healthy food in any environment, for example, home, school or the wider community setting, including retail outlets [18]. Ultimately, the food available at local levels reflects a country’s overall food supply. Policies that determine the food supply in NZ sit within a broader ideological framework of neoliberal capitalism [19]. From 1984, successive governments followed a policy framework, designed to limit state regulation, and increase trade liberalisation. The NZ economy relies on the production of high-quality foods for export, especially milk and dairy products, beef, lamb, and fruits and vegetables [20]. However, NZ imports large quantities of discretionary food products, including sugars, biscuits and chocolate [20]. In 2019, over half (52%) of the packaged foods available in NZ supermarkets were discretionary foods, and over two-thirds (69%) had been ultra-processed, with added sugar, salt, and fat [21].

Public health nutrition policies that impact the right to food in NZ have changed over time. From 1999 to 2008, there was a comprehensive nutrition and physical activity policy, Healthy Eating Healthy Action (HEHA) [22], based on the Ottawa Charter [23] and aligned with He Korowai Oranga, the Māori health strategy [24, 25]. In 2007, food available for sale in schools was regulated [26], however this ceased in 2008 [27] and HEHA was cancelled in 2011 [25] and replaced with ten Healthy Families programmes [28] and a Childhood Obesity Action Plan [29, 30]. In 2019, a ‘Healthy Active Schools’ programme was introduced to support schools to increase physical activity and introduce healthy food policies [31].

NZ does not have a tradition of food provision in schools. In lower decile schools, a combination of charitable and government/corporate programmes have provided breakfast, fruit, and in some cases, lunches [32]. In 2020, government food provision of lunches was trialled for the first time to some schools as part of a broader aim of the current government to reduce child poverty [33]. It was recently expanded in response to the economic effects of the Covid-19 pandemic [34].

Modelling research using NZ data has quantified the potential health impacts of fruit and vegetable subsidies, taxes on saturated fat, sugar, salt and junk food [35, 36]. There is also well-established evidence for the potential health impact of a tax on sugar-sweetened beverages [37]. However, successive NZ governments have been reluctant to introduce policies based on public health evidence. Government policies have tended to align with the food industry’s interests, especially in the absence of regulation [38]. For example, there has been no taxation on unhealthy drinks/foods, no government regulation of food marketing, and since 2008 no requirement of schools to regulate foods [39]. There is also no local government regulation of food supply within an area. This situation can be described as an example of policy inertia resulting from the combined effects of inadequate political leadership, industry opposition to effective policies and public apathy [8] .

How a problem is represented or framed determines possible solutions [40]. A rights-based approach defines the responsibility of governments as a duty-bearer to uphold citizens’ rights [41]. Health inequities between Māori and non-Māori, including obesity and food insecurity, are evidence of the Crown’s failure to uphold indigenous rights [42]. This is a breach of NZ’s founding document, the Treaty of Waitangi [43]. The Treaty is a partnership agreement signed in 1840 between Māori and the British Crown that ultimately enabled the establishment of government in NZ, and guaranteed to Māori, equal benefits of citizenship. This places responsibility for Māori health equity on the NZ government [25, 31].

This study aimed to investigate policy options that would safeguard the rights of indigenous children to healthy food. It does so through a NZ case study focused on the rights of tamariki. Analysing the food available to children through a rights-based approach enables the analysis to move away from victim-blaming discourses, which are often used in relation to indigenous peoples, including Māori [44], to one that considers structural issues that shape food availability.

## Methods

This research is positioned within an overall framework of Kaupapa Māori research (KMR) theory [45] and is explicit in its aim of improving Māori health. KMR theory emerged from Māori academic critique of research that gathered information from Māori communities and then framed Māori in deficit when compared to non-Māori, without providing either the historical context or any solutions to perceived problems. A primary focus of KMR is also to develop and advance as Māori using Māori knowledge, values and processes [45]. The CONSIDER statement provides a checklist for best practice research involving indigenous peoples [46]. This study aligns with many checklist items in that the research priorities emerged from a Māori researcher working with a Māori advisory group, who informed the overall research design. The lead researcher is Māori (CM), supported by a senior Māori academic (CL). In addition, the KMR theoretical framework used is informed by social justice approaches, where the structural causes of health inequities are explored [47]. Therefore, this research focused on policy rather than ‘downstream’ individualised solutions. The views of Māori health experts in this field were explicitly sought to privilege Māori voice within the research [47].

Fourteen in-depth interviews were held with 15 key stakeholders, two were interviewed together. A purposive sample was used, and participants were identified based on the researchers’ knowledge and by using a snowball technique, where each participant was asked to identify other key informants. The range of people interviewed included Māori participants from academia (*n* = 4), a Māori health provider (*n* = 1), a public servant (*n* = 1) and a staff member of a district health board (n = 1). Views of non-Māori, who had experience in working in this area, were also sought, and people interviewed came from the following backgrounds: academia (*n* = 1); public service at either government or local DHB levels (*n* = 5); and a representative with experience in local government issues (*n* = 1). Stakeholders were selected for their experience and expertise in food and nutrition policy, research or practice. The views of stakeholders representing other sectors related to this policy area, for example, food industry representatives were not sought as their views have already been well documented within a New Zealand context [48]. Instead, this research specifically sought Māori perspectives which have not been explored in-depth related to this topic. Participants were asked if they would like to remain anonymous or be identified in the written analysis. The people interviewed from an academic background were comfortable with being identified. However, all bureaucrats interviewed needed to remain anonymous. Therefore, in the written results, all participants’ anonymity is preserved.

This study is part of a broader mixed-method study exploring food availability for tamariki. Therefore, before the interview, participants were given a background information sheet with initial research findings from a related project by the researcher about food availability and tamariki in order to provide context. This is available in the research protocols section of the following link: https://www.otago.ac.nz/heppru/research/index.htm [49]. The information summarised research related to food environments for NZ children, for example, that food retailers are a prominent destination for NZ children [50], and unhealthy food is widely available on the journey to school or in convenience stores [51]. A related area is that NZ children are exposed to high rates of unhealthy food marketing, reflecting limited regulation [52].

A semi-structured interview outline was developed to guide the interviews. Interviews were conducted either face to face (*n* = 4) or by ‘zoom’ video-conferencing (*n* = 10), and were recorded and then transcribed. Topics covered in the interviews included: the right to healthy food from a Māori perspective and the feasibility and acceptability of a range of policy options to increase healthy food availability for tamariki, primarily focusing on the food available in communities. The research examined policy options that would decrease the availability of unhealthy food for tamariki (interview prompts included taxation of sugary drinks and regulation of local food environments, e.g. green zones around schools to limit unhealthy food outlets). The research also explored policy options that would increase the availability of healthy food for tamariki (interview prompts included food subsidies, food provision or community food initiatives, e.g. food cooperatives and gardens). The feasibility, acceptability, barriers and implementation aspects of each policy were explored.

In-depth interviews are a conversation with a purpose and, as with all communication, require a degree of rapport between the interviewer and interviewee [53]. Rapport was established in the interview informed by the hui process [54] where the conversation follows stages in line with Māori protocols with an emphasis on establishing a shared connection before asking about key research questions. The interviewer (CM) had worked in the discipline for several years. She was already known to many of the participants, which enabled access to, as well as rapport with, stakeholders [55]. The interviews were transcribed and coded using thematic content analysis [53]. CM familiarised herself with the data, by listening and re-reading each interview transcript at least twice, and then identified key themes which came from each interview and grouped these into codes. The entire data set was coded and analysed. Attention was given to whether the data emerged from a Māori or non-Māori participant, to retain the Māori voice in keeping with KMR methodology. CM engaged LS in analytical conversations to discuss the research themes. An iterative reflexive approach to the analysis identified inductive themes which were drawn from the data. These related to participant views on policies to improve healthy food availability for tamariki [53]. Deductive themes that are themes driven by a researchers theoretical interests, were also identified [53]. These were informed by the theoretical concepts of KMR and literature on rights-based approaches. Further refinement of the themes progressed as part of the writing process, and in discussion and peer review with all members of the research team. Quotations as data are presented in this paper to illustrate the themes. Participants’ details are presented by the relevant quotation using a code that identifies their ethnicity, e.g. MP1 - Māori participant 1, NMP1 – non-Māori participant 1. Ethical approval was received from the University of Otago Human Ethics Committee Ref D 18/300.

## Results

The following themes on ways of ensuring the rights of tamariki to healthy food were identified from the analysis of transcripts:

- A comprehensive policy response that supports children’s rights;
- Ending child poverty to enable the right to food;
- Food provision and food policy in schools;
- Local governments to improve healthy food availability;
- Māori voices and values in decision-making.

These themes are discussed in the following sections.

### Theme 1: comprehensive policy response that supports children’s rights

Participants recognised that the right to food was a key issue impacting Māori. This issue has been previously identified by Māori [25], with one Māori participant commenting they developed their research interest in this area as a result of discussions they had with the Māori community.

*I went to the rūnanga (tribal committee) there... and I asked them what’s the most pressing health issue in the rūnanga in the Hawke’s Bay region, and they said that its access to food, straightaway, there was no hesitation, you know it wasn’t diabetes, it wasn’t heart disease. (MP1).*

The majority of participants conceptualised the issue as an outcome of a broader systemic failure. One participant discussed a lack of access to healthy food as resulting from systemic factors impacting Māori.

*The real factor is that, because of colonisation, coloniality, racism, capitalism, Māori children and young people do not have access to healthy food and water, like they actually don’t because it’s been taken away from them. (MP2).*

Māori participants spoke of the impact of colonisation on Māori, raising the example of environmental pollution, which had diminished access to traditional food sources.

*Thinking about whenua (land) and whakapapa (ancestral connection to environment), recognising that we’ve actually created the environment that makes it really difficult for people to have access to food sources that would have been more healthy, and even when they do have access to those things, like in Waiwhetu watercress, it’s like but the stream is so polluted. (MP3).*

Although Māori participants were more likely to frame the issue in terms of an outcome of colonisation, all participants recognised the complexity of the problem of food availability. There was no one policy solution that all the participants suggested would solve the problem. Instead, all participants emphasised that a range of policies would be needed.

*There is only one thing we need to do and that’s everything. (NMP1).*

Many participants discussed improving environments generally for children, as a starting point, before implementing specific policies related to healthy food and drinks.

*We expect a lot from children, who are in a much more constrained environment in terms of their ability to exercise their sovereignty over decisions about what they eat, and how much money they have, or even where they are, … I would think things that we can do at the level of, making environments more generally health protective for children, before we get into any sort of individual taxes or anything. (MP3).*

Specific to nutrition policy, many participants reflected that the previous Healthy Eating Healthy Action policy [16] was an example of a broad policy approach.

*There is never any one action that government or anyone can do that’s going to solve the problem, it is a complex problem and needs a comprehensive response and so something like HEHA was an attempt at a comprehensive response which obviously got nipped in the bud prematurely.* (*NMP2).*

Reflecting on the disestablishment of HEHA and the lack of evidence-based policy to reduce obesity, many interviewees were very aware that the introduction of any policies to reduce the availability of unhealthy food would come up against industry pressure. Most were aware that broader economic considerations motivated government policy.

*Make changes to policy but being hamstrung really by this need by our governments, both National and Labour, to have economic growth at all costs. We can’t...possibly do anything that might harm small businesses … like convenience stores … as if we care more about businesses that sell crappy foods than we do about the population’s health. (MP5).*

As a result, many were very pessimistic about the introduction of any policies that might regulate unhealthy foods and drinks.

*Interviewer: What would it take for, for those kind of policies to become politically.*

*feasible?*

*MP5 I suppose when they can no longer provide dialysis to people with diabetes.*

However, some participants discussed that aligning the issue of the right to adequate food and food availability within broader policy platforms such as wellbeing or environmental sustainability could be an opportunity to influence policy change.

*I think climate change is a pretty strong momentum, but you know also actually within this government, they have been pushing … a child poverty, equity wellbeing agenda, so that is also a political opportunity, so … you have to create a collective narrative that is encompassing of those things, so the people at a community level, who really care about the sustainability of the food environment, or really care about pesticides, or really care about the lack of bees, or really care about school food, … or really care about you know healthy food availability and so on, what’s the common narrative that is going to unite them, so they’re all pulling in the same direction, so that that civil society voice can lift up, and demand more policy action. (NMP1).*

However, despite acknowledging that a comprehensive policy response was needed, of immediate concern for all participants was the issue of food insecurity and the need to support whānau to access food.

### Theme 2: ending child poverty to enable the right to food

All participants argued that addressing child poverty and increasing income for families was essential for upholding children’s rights, especially the right to food*.* They acknowledged the barriers many whānau Māori (Māori families) face in terms of accessing healthy food. They also recognised that policy change needed to be at the level of supporting whānau, rather than just focused on children as individuals.

*If we were to consider the rights of the child first and foremost, we would be making sure that their families have sufficient resources to be able to love and care for them and I think many, many families in NZ don’t have, even if they are working … don’t have enough of the essential resources. (MP5).*

Participants saw the issue of addressing poverty as the main issue determining the right to adequate food for tamariki. In discussing income levels, many participants highlighted the broader determinants of health that include housing, employment and wages.

*I actually think there are bigger more systemic problems around you know housing and employment, and wages and you know, life being affordable for families on really low incomes, and working …, I’m very passionate about food don’t get me wrong, and we can do as much work as we can in the food industry space, but making food better quality, and making a lot of it more affordable, isn’t going to resolve the problem that the family might have $60, to feed 5–6 people for a week, because they’ve had to put so much into housing, and they’ve got to heat the house, and all these other bills and expenses …, I don’t think it’s a simple problem.* (*NMP3).*

Many participants interviewed had experience with research or community programmes to alleviate food poverty. For example, those interviewed working in health promotion organised a food cooperative, one was a board member of a community programme based on school food gardening and food provision and one researcher had carried out a food box research programme with Māori. This participant described the real struggle for whānau in low paid employment to afford food.

*Amazingly enough, they just can’t afford food, they can’t afford food because the housing, the rents are so high, their family dynamics change a lot too, there’s transition within families, so it’s really difficult to keep a steady hand on the, on the budget for the household. (MP1).*

This participant’s research had tested a fruit and vegetable box delivery to low-income whānau. While the whānau consumed the food delivered for no-cost as part of the research study, the ongoing sustainability of the programme was impacted by cost.

*I wanted to test that kind of part out, so whether a fruit and veg cooperative would work, in this situation, and we found about on average 5 families a week would purchase the $5 bags, most of them said they just couldn’t afford it. (MP1).*

Other participants involved in food cooperatives or other community programmes also commented that the sustainability of their programmes was supported with funding from the District Health Board (local government health agency) and philanthropic sources, as well as voluntary labour. In one example a family box of fruit and vegetables for NZ$12 a week was available to approximately 1000 families through a region. Similar to the previous example, the cost barriers were discussed:

*The buying system which is usually paid in advance, the $12 you know sometimes the lower-income find that challenging.* (*NMP4).*

Participants were able to comment in detail about some of the challenges faced in running food cooperatives, which apart from pricing also included the familiarity and acceptability of what was included in food boxes to participants. Due to their experience in working in this field, many participants also questioned the narrative of individual food choices, suggesting that this is a problematic assumption given their low income.

*We talk about people knowing how to budget, these people know how to budget because they’ve got, they have to, they don’t have any choice in this matter, you know they don’t have a choice, they don’t have a choice to give their children healthy food, because there is no choice when you’ve got no money.* (*NMP3).*

Many participants described food access issues in low-income urban areas. One participant described their work with children from a low decile area on the outskirts of Wellington. They said many of the children in the study had not travelled into the central city of Wellington, so were very reliant of what was available locally in their area. This tended to be convenience stores and takeaway outlets. The potential of a rights-based framing was explored with participants. It was seen as a mechanism to advocate for an environment that is more health protective:

*I always think in that environment where you’ve got, poverty real poverty and a real divide between wealth and poverty, your ability to actually make choices with the money that you have, are really constrained. So I think, … which does bring it back to rights, … what we’re prepared to tolerate as the food environment for our children as a society, and then blame them for making poor choices. (MP3).*

There was an understanding by most participants that cheaper, unhealthier food was often the only option. While there was some support among participants for the introduction of a sugary drinks tax, most participants were concerned that when taxes were raised as a policy option, for example, a sugary drinks tax, that this would impact Māori. One Māori participant termed it a ‘tax on Māori’. Many suggested that any form of regulation that would increase the price of food would need to be counterbalanced by policies that would decrease poverty and increase the availability of healthy food and drink.

*I think taxing will be good but then you haven’t really addressed the actual thing about well what are they going to have instead, and if people say oh water, ok well that’s good if you’re not in a rural place, that relies on rain … I lived in a rural place that didn’t have, so you relied on a water tank, so if it didn’t rain there was no water, and if there’s no water and you can’t afford to buy, so you know poverty isn’t just about eating food, it’s about drinking, so the only time that I could drink water was literally at the school. (MP2).*

Many suggested they would start with economic policies, for example, ensuring the living wage or increasing benefits.

*When people call for one thing like say oh let’s have a tax on sugar I mean it may or may not work, …, working where I do at the moment it is not politically palatable so it’s a bit of dialogue, … even if it was, it’s still is not the answer, it has got to be all these other things and we’ve got to be looking at a living wage or a benefit that enables people to live and make the choices that other people take for granted*. (*NMP2).*

Beyond these broader economic policies to support income, one of the key policy options discussed with participants that could impact the right to adequate food was food provision in schools.

### Theme 3: food provision and policy in schools

Over the time that the interviews were carried out, the government introduced a food provision trial in low decile schools. This was generally seen as a very positive policy for tamariki.

*If you had food in schools that was really healthy, free food in schools, then to me that feels like a good counterbalance, to decreasing the availability of unhealthy food, because then I’m not worrying that actually people are hungry, because if they’re hungry they’re going hungry, if they’re really hungry I’m not going to judge them for eating a bag of twisties, but so if you can, I mean I guess that policy, and it also removes sort of some of the barriers and stigma, around if you just make healthy food available. (MP3).*

The importance of food provision by the government rather than charities was seen as necessary. Two participants who worked with schools suggested that lunch provision provided by charities could be unhealthy as there tended to be a reliance on packaged rather than fresh foods. Another participant commented that children singled out to participate in food provision programmes by a charity ran the risk of stigmatisation, therefore food provision needed to be universal within a school. Food provision was also seen as a way of decreasing the burden on teachers.

*Anecdotally a lot of teachers just make extra lunch to give to kids, .. from their own pocket, bring loaves of bread or whatever. My mum, she’s a principal now she was a teacher, so she used to do that, she’d pack my lunch, my sibling’s lunch and then extra lunches, to give out extra sandwiches, unfortunately, because it’s coming out of the teachers pocket, it would often be noodles, packets of noodles, the cheap food, anything to fill a kid up that didn’t have lunch that day. And I saw that a lot when I was working in schools as well, noodles and things. (NMP6).*

While participants were supportive of the introduction of food provision in schools, the details of the programme were of interest, especially to those participants with a background in nutrition.

*I think it sounds like it’s been set up as a bit of an experiment, and that there’s going to be different ways that it’s delivered, in different schools in the different areas, whether the school itself is providing the food, whether it’s going to be brought in from an external provider, and I think us in the nutrition world are all interested to see … how it’s made healthy, what guidelines its aligning to and how that’s going to be enforced, and how practical it is for schools.* (*NMP5).*

Some participants discussed the government’s policy ‘Healthy Active Learning” which was introduced over the time of this study. Reflecting on the voluntary rather than compulsory nature of the programme, participants suggested that there may be potential to increase health inequity, with the least deprived schools more likely to be able to implement healthy food policies. Another participant who had been in a governance role at their children’s school discussed the time and resources it took to introduce a food and nutrition policy. They suggested a compulsory policy implemented within schools would be less time consuming than each individual school developing their own policy. However, participants were aware that the implementation of a healthy food policy within a school might have the potential to cause unintended harm.

*I think we need to be really careful that we are not increasing inequities by saying only bring healthy kai (food) … for some that would mean no kai comes to school. (MP6).*

The issue of balance for both the provision of healthy food with the restriction of unhealthy food was also raised in this context.

*How you balance for our whānau schools being places for healthy kai, but at the same time not penalising those that may not have the means to be able to bring in that type of kai, so those penalties for people by looking through lunchboxes. (MP2).*

Rather than targeting food that was brought to school in a child’s lunch, participants were more supportive of policies which dictated what food items were for sale in a school. Many people remembered the National Administration Guideline (NAG) to regulate the sale of food in schools and regretted that this policy was disbanded.

*I can remember that I was in college then, I can remember it all getting pulled from the canteen, no more chocolate bars, no more ice blocks, no more lollies, fizzy and everyone was annoyed, and that blew over and then I started working in it, and then it was all back in again, I was like oh no, you’ve done it, why take it away? (NMP3).*

One participant discussed in detail the development processes that had gone into the NAG at a Ministry level.

*What had happened was that the government said we are going to make it a requirement that only foods that are fit for everyday consumption and should be part of a healthy everyday diet will be allowed to be sold or provided in the school. So, that was everything – catering, fundraising, sausage sizzle – and all those things that they had to do. And there was this whole pile of work that the Ministry did around creating those guidelines around what you could and couldn’t sell in the school. (MP7).*

Interestingly this participant suggested that food industries who had reformulated products to fit with the planned guidelines had been disappointed when the NAG was disbanded because they would no longer be selling products in a closed market. Beyond the school gate the issue of the widespread availability of unhealthy food within commercial areas close to a school was also raised by participants. Many questioned the narrative of parental control of a child’s food ‘choices’.

*I have very little control over what he does after school, even if he doesn’t take money with him to school his mates will have money and they hang out at the shops after school buying sugary drinks and … lollies and pies and chips and things like that so you know people might say oh that’s up to parents to control their kids but we actually, you know … don’t have any control over that. (MP5).*

As the environment impeded parents’ control over what their children were consuming, participants argued for the need for communities and local governments to have greater control over the food outlets in a specific locality.

### Theme 4: local governments to improve healthy food availability

Another major theme discussed by many participants was the food available for children within their local environment. This was in part, prompted by one of the suggested policies for enhancing healthy food availability - to develop a green zone around a school where there is a limit on the density and proximity of food outlets. Many participants thought this would be a useful policy initiative.

*I want to see how we can empower the local government to be better supported to regulate how many junk food shops are available but are readily accessible by all children. I mean wealthy people ... wouldn’t choose to live in a neighbourhood that was surrounded by convenience stores and take away shops and things like that … so why is it good enough for some children to live a lovely safe, healthy food environments that enhance their right to good health but it is not good enough for all children. … if a shop is dependent on making a profit from selling food that makes us unhealthy well then … why is that an allowable model? (MP5).*

Another participant saw the lack of investment in low-income neighbourhoods as a value judgement on who is deserving and who is not.

*I see it as a system of oppression, where some people are more deserving of you know, good healthy environments than others. (MP2).*

Those that worked in health promotion also discussed their frustrations with the food environment surrounding schools. One described her work to support schools to have a water only policy being negated by the availability of sugary drinks within the local environment. The mechanisms to introduce a ‘green zone’ around a school was explicitly discussed with a key informant from the local government sector. They said that currently;

*There are no powers for Councils to control the number of shops, in any kind of area*. (*NMP7).*

This is because the Resource Management Act, which is the legislation that NZ councils currently use, is not a tool for planning. Instead, it merely manages the effects of activities on the environment. Therefore, most activities are allowed as long as there are no negative impacts on the environment, which this participant suggested is interpreted narrowly rather than broadly. This participant then discussed the potential of National Policy Statements that apply to all local authorities as a mechanism for introducing legislation that would limit unhealthy food outlets. They noted that when the RMA was introduced in 1991, there was;

*An expectation that central government would develop national policy statements. (NMP7).*

However, in their opinion, this mechanism had been underutilised. Beyond the role of regulation the discretionary capacity of local governments to support local food initiatives, for example, local fruit and vegetable markets, or local food gardening initiatives, was also highlighted by participants. The capacity for local solutions was highlighted by one participant who is a board member for a local-based food project. This project started as a garden project using land in a school to provide food for school lunches and teach children about gardening and food. This has grown to support other projects led by the community, for example, a local community café, and small businesses. This participant saw community lead projects as part of the solution to improving food availability at local levels:

*I think it’s [legislation] an important piece of the puzzle, but it’s very top-down, and I am not sure that it’s … enabling communities. It is part of the picture. I guess one of the things for me that I have learnt and I feel like I am learning is that communities do have their own solutions and there are amazing people within communities that given the support, will lead to change. (NMP8).*

Although there was support for community-led gardening programmes, with one Māori participant noting that these were an expression of food sovereignty, participants were acutely aware not to ‘romanticise’ gardening or promote it as a single solution. Of more concern, especially for Māori participants, was the inclusion of communities and especially Māori, in local decision-making.

### Theme 5: Māori voices and values in decision-making

In discussion about local governments, Māori participants argued for Iwi (tribes) to have an increased role in a local community environment.

*I do think that iwi (tribal) role isn’t just about the little bits and pieces that the Iwi (tribes) do, but actually what is the iwi role in that they have expectations for our whenua (land), our wairua (spiritual values), our tikanga (customs), our communities … And actually, we demand, for example, that we be consulted on [food] outlets going into our communities and servicing our people.* (*MP7).*

Other participants focused on the participation of children in local government processes.

*So Porirua Council … they’ve actually got a children’s board or a children’s panel, and I think they have some involvement in decision-making around, I’m not sure at what level, … so imagine if you had children and Māori, in … Council making collective decisions about food environments, … that would be awesome, … real optimisation of tino rangatiratanga (sovereignty), if Māori could as Iwi (tribes) have, or hapū (subtribes) or, have some sort of more meaningful input into making decisions about environments. (MP3).*

Participants conceptualised an increased role for Iwi within local government structures within the framework of the Treaty of Waitangi.

*Ideally if you had a Treaty-based sort of partnership, or if District and Regional Councils operated in that sort of Treaty-based way, then Māori or Iwi would have much more of a say, in sort of the regulation of those things. (MP3).*

Māori participants often referred to Māori values about food arguing for Iwi to be included more in policy-making at local levels because Māori constructs of thinking linked issues together using overarching concepts and values. One participant described the example of a Māori cultural performing arts event, where the local Iwi had been funded to look after the wellbeing of people in any way they chose. The Iwi had focused on the value of manaaki (caring for people) and expressed this through the following ways:

*So, in terms of the manaaki manuhiri (caring for visitors), they had all the local growers provide big tubs of fruits and vegetables to the marae (cultural centres) who were hosting to make sure there was lots of fresh produce available to the marae. Plus, Kahungunu (local tribe) at that time put a whole pile of money into the marae to make sure they were ready to host. And then manaaki tangata, (caring for people) it was alcohol-free, drug-free, fried food-free, sugar-free wherever they could; so, they really went hard out. Manaaki tangata meant that those things didn’t have a place. And then they also had this manaaki whenua (caring for land), so they had para-kore (zero-waste) aims. So, if you were selling something there to eat, it either had to be compostable, recyclable to try to minimise waste and they had fully manned composting stations where compost goes here … So, what they showed us was that when you change the construct and it is no longer a policy or a rule, but it’s about the expression of manaaki …*. N*ow that doesn’t just happen at Matatini (Māori performing arts festival), that happens at anything Kahungunu hosts, that’s the way they behave. It’s not about a don’t or a do message, it’s about the being of us as Ngāti Kahungunu that is the expression of Ngāti Kahungunu. So, it’s a policy that’s not a policy if you know what I mean, it’s just a way of being.* (*MP7).*

Māori values such as manaaki offer a way of thinking about the complexities of the issue of food in a single narrative that bind together the separate elements of the issue. Limited healthy food availability is ultimately an outcome of colonisation, therefore the solution lies in a decolonising approach where Māori voices and values are central within NZ policies and policy making processes that influence food availability and enhance the right to adequate food.

## Discussion

This paper considered the right to adequate food for indigenous children through a NZ case study. It explored stakeholders’ views of policy options to ensure the right to food and health for tamariki. The discussion focused on policies that could increase healthy food availability and decrease unhealthy food availability. The broad research question yielded an equally broad range of views about policy from the stakeholders interviewed. The findings of this research suggest that to ensure the rights of tamariki there should be: a comprehensive policy response that supports children’s rights; child poverty ended to enable the right to food; food provision and food policy in schools; stronger use of local government policy-making processes to improve healthy food availability and Māori voices and values strengthened in decision-making for this issue.

Theodore et al. (2015) stated that to reduce the burden of obesity for Māori, the NZ government must prioritise the creation of healthier food environments by addressing the cost and availability of healthy foods, and reduce the socioeconomic disparities that drive obesity [25]. Our findings are consistent with their analysis. Previous research with Māori and other indigenous communities has described food insecurity and obesity as interconnected issues [56–59], ultimately linked to structural factors including racism, colonisation, and the associated nutrition transition, which continue to result in inequities [60, 61]. The right to adequate food for indigenous people has been recognised as a collective right that is multi-dimensional and interconnected with rights to culture, land, and resources, and to self-determination and non-discrimination [62].

Our research focused on the right to adequate food as a critical issue rather than obesity. This was informed by discussions with Māori stakeholders and also influenced by Bacchi’s work on problem representation, which argues that possible solutions are determined by how an issue is framed [40]. We suggest that obesity is not the problem. Obesity is an outcome of child poverty and a lack of political commitment to the right to adequate healthy food. The problem identified in this research is that all tamariki do not have their right to healthy food realised. Although previous research on obesity has included equity considerations, equity was the central issue in this study. Ending child poverty to enable the right to adequate food was a central theme. NZ markets itself as one of the best countries to bring up children [63], however this is simply untrue as over a quarter of its indigenous children experience food insecurity [17]. Our findings indicated support for policies that ensured whānau had enough income to feed their children, for example increasing the minimum wage to a living wage, and increasing state welfare provisions. Food provision within schools was strongly supported. It is striking that NZ has not had food provision in schools unlike many countries it compares itself to, for example the UK and USA. Countries, such as Brazil, used universal food provision in schools with different strategies, including strong political leadership, to ensure the right to adequate food [64]. Food provision was seen as a means of balancing more restrictive policy options such as taxation. Related research has found that the NZ public and stakeholders are supportive of a subsidy on fresh fruit and vegetables, but less supportive of increasing tax on foods high in fat, sugar, or salt [65, 66].

NZ research has identified the food industry influence on policies [67], and industry framings of obesity [48] as a problem of individuals making unhealthy ‘choices’. This narrative is especially stigmatising to Māori, given the economic barriers to food. Other researchers have also noted that neo-liberal constructions of food ‘choice’ are particularly shaming for whānau experiencing either obesity or food insecurity [68–70]. There has been a tendency in the NZ media, to take a victim blaming and racist approach to how obesity and food insecurity are framed and therefore solutions promoted tend to be focused on individuals rather than upstream structural factors [71]. This framing has also contributed to public apathy and a pattern of policy inertia from successive NZ governments. In contrast, this research has emphasised that a broad ‘whole of government’ response across multiple domains is required. This is consistent with research in NZ on stakeholder views of the effectiveness of NZ government policy to prevent obesity [72, 73].

A strength of this research is that it prioritised several aspects of responsiveness to indigenous people as identified by the CONSIDER statement [46]. Aspects included the overall research question identified with Māori input, the KMR theoretical framework and the resulting analysis focused on the structural inequities of food availability. The need for Māori voices, (including children) in decision-making, especially but not limited to local levels, was a key finding specific to this research. This finding can be broadened to other indigenous peoples, whose voices tend to be excluded from policies. The right of children to be involved in decision-making is recognised in UNCRC as well as the collective decision-making process used by Māori [1, 43]. While there is limited research in NZ, one study related to children’s perceptions of food marketing and what to do about it, found that children were able to suggest credible solutions to address issues of concern [74]. There are also recent NZ examples of school children advocating for water only schools [75]. Future research could comprehensively explore this issue with tamariki themselves, modelled on examples from the UK where children’s voices have been included in action research on the future of the nation’s food [76].

There are currently limited mechanisms for local government to influence food availability, and introduce policies, for example green zones around schools as in South Korea [73]. There is one recent example of local community advocacy to prevent a fast-food outlet opening [77], however this is the exception to the current status quo. Therefore the potential of a national policy statement by government to enable local governments to restrict unhealthy food outlets need to be further explored. A recent RMA review has suggested changes to enable increased Iwi involvement in processes consistent with the Treaty of Waitangi [78]. If implemented, Māori values could be included and reflected within policy regulating local environments. Māori values such as manaaki offer a way of thinking about food using a system based framework that connects the rights of tamariki and the environment [79, 80]. The dramatic changes to the economy and the policy making environment globally due to the COVID 19 pandemic, allows a policy reset that prioritises the right to adequate food for tamariki.

## Conclusions

This research canvassed the issue of the right to food and availability with academics and policy makers as an initial case study. Findings from this research strongly support that NZ is failing in its duty to provide the right to adequate food for indigenous children. Given the importance of food to health, the need for a broad policy approach from the NZ government to safeguard the right of tamariki to adequate food is urgent. This includes economic policies to end child poverty, as well as specific strategies such as food provision and food policy in schools. Within this policy approach, Māori voices and values need to be central to the process.

## Data Availability

The dataset used and/or analysed during the current study are available from the corresponding author on reasonable request.
